# Histone Deacetylase Inhibitors (HDACis) as Latency-Reversing Agents in HIV Cure Strategies: Chemistry, Selectivity, and Clinical Perspective

**DOI:** 10.3390/v18060673

**Published:** 2026-06-16

**Authors:** Hanan Almolhim

**Affiliations:** Department of Chemistry, College of Science, Northern Border University, Arar, 91431, Saudi Arabia; hanan.almolhim@nbu.edu.sa

**Keywords:** HIV, latency, histone deacetylase inhibitor, latency-reversing agents, shock and kill, antiretroviral therapy, structure–activity relationship, isoform selectivity, reservoir eradication

## Abstract

Latently infected resting CD4+ T cells represent the principal barrier to HIV eradication. Their immunological quiescence renders them invisible to combination antiretroviral therapy (cART) and host immune surveillance, sustaining a reservoir that mandates lifelong treatment. The ‘shock and kill’ strategy seeks to reverse this latency using pharmacological latency-reversing agents (LRAs) so that immune effectors and cART can eliminate reactivated cells. In this narrative, structured review, we examine histone deacetylase inhibitors (HDACis) as LRAs from a medicinal chemistry perspective. The review places particular emphasis on structure–activity relationship (SAR), isoform selectivity, and the mechanistic basis of differential clinical performance, synthesizing evidence from preclinical, ex vivo, and clinical studies published between 2010 and 2026. Four structural classes of HDACis—hydroxamic acids, benzamides, cyclic depsipeptides, and short-chain fatty acids—differ substantially in isoform selectivity, potency, pharmacokinetics, and tolerability. No single agent has achieved statistically significant reservoir reduction in clinical trials, highlighting the apparent inadequacy of the ‘shock’ phase alone and suggesting a need for complementary ‘kill’ strategies. Rational design of HDACis informed by isoform-selective SAR, combined with emerging combination LRA strategies and immunological ‘kill’ components, represents a promising direction toward a functional HIV cure, though substantial translational hurdles remain.

## 1. Introduction

Human Immunodeficiency Virus (HIV) remains a defining global health challenge. According to the WHO 2024 Global HIV Update, approximately 40.8 million people are living with HIV, with 630,000 HIV-related deaths recorded in 2024 [1]. Combination antiretroviral therapy (cART) has fundamentally transformed HIV management, reducing plasma viral loads to undetectable levels in adherent patients [2]. Yet, a definitive cure remains elusive.

The central obstacle is the HIV latent reservoir, which is a population of long-lived, transcriptionally silent, resting CD4+ T cells harboring integrated provirus. These cells are not eliminated by cART and can seed rebound viremia within weeks of treatment interruption. The reservoir is seeded early in infection, is replenished during episodes of intermittent viremia, and exhibits substantial clonal expansion, which is a phenomenon increasingly recognized as a major barrier to eradication [3,4].

The ‘shock and kill’ strategy, proposed to overcome this barrier, uses LRAs to reactivate latent virus (‘shock’), rendering infected cells susceptible to immune-mediated clearance or cytopathic effects (‘kill’), while cART prevents de novo infection [5,6]. Several LRA classes have been investigated, including protein kinase C (PKC) agonists, BET bromodomain inhibitors, DNA methyltransferase inhibitors, and histone deacetylase inhibitors (HDACis). Among these, HDACis have received the most clinical attention owing to their well-characterized epigenetic mechanism, structural diversity, and prior regulatory approval in oncology.

Critically, however, no clinical trial to date has demonstrated that any LRA, including HDACis, achieves a statistically significant reduction in the size of the latent reservoir. This gap between laboratory promise and clinical efficacy is the central unresolved tension in the field, and it motivates the analytical focus of this review.

Why This Review?

Several reviews have cataloged HDACis activity in HIV [7,8]; however, the literature since 2020 has produced critical new insights that alter the interpretation of earlier clinical data: (i) identification of intact versus defective provirus populations has revealed that most reactivatable proviruses are transcriptionally defective and therefore incapable of contributing to viral rebound [9]; (ii) clonal expansion of infected cells can maintain reservoir size even when individual proviruses are cleared [10]; and (iii) HDACis may paradoxically impair the CD8+ T cell killing activity required for the ‘kill’ phase [11]. This narrative, structured review, which does not follow a systematic review or meta-analysis protocol but applies a structured search strategy and pre-specified inclusion criteria, integrates these insights with a chemistry-focused structure–activity relationship (SAR) analysis to provide an updated framework for rational HDACis development. It is distinguished from prior reviews by its explicit cross-class SAR comparison and its engagement with post-2020 mechanistic data that reframes the clinical evidence base.

## 2. Literature Search Strategy

A structured literature search was conducted across five databases. The search strategy, inclusion/exclusion criteria, and results are summarized in Table 1.

Titles and abstracts were screened manually by the author according to relevance to HIV latency reversal, HDACis medicinal chemistry, and clinical translation. Full texts of potentially eligible articles were then retrieved and assessed against the inclusion criteria. Reference lists of key reviews and clinical studies were additionally screened to identify relevant articles not captured in the database searches.

## 3. HIV Latency: Molecular Mechanisms and the Reservoir Problem

HIV latency is established when the viral genome integrates into the chromatin of a host cell and becomes transcriptionally silent. Although resting CD4+ T cells represent the dominant and best-characterized latent reservoir and are the primary focus of this review, HIV also establishes latency in other long-lived cell types, including macrophages, monocytes, microglial cells, and possibly hematopoietic stem cells, each of which may contribute to reservoir persistence and complicate eradication strategies [12,13]. This silencing is maintained by multiple, overlapping epigenetic mechanisms: (i) deacetylation of histones H3 and H4 at the HIV long terminal repeat (LTR) promoter by recruited HDAC1, HDAC2, and HDAC3, promoting chromatin condensation; (ii) DNA methylation of CpG islands flanking the LTR; (iii) sequestration of the positive transcription elongation factor b (P-TEFb) by HEXIM1; and (iv) absence of key transcription factors (NF-κB, NFAT) in resting cells [14,15,16]. Mbonye and Karn (2014) provide a comprehensive framework for understanding how these cellular signaling pathways converge on the LTR to maintain silencing, emphasizing that effective latency reversal must engage multiple nodes simultaneously [16]. Coiras et al. (2009) further contextualize the evolutionary basis of latency as a viral immune-evasion mechanism [17].

A critical and recently appreciated complexity is the heterogeneity of the latent reservoir. Deep sequencing studies from the Siliciano laboratory and others have demonstrated that the vast majority (>90%) of proviruses in patients on suppressive cART are defective, carrying large internal deletions, APOBEC3G-induced hypermutations, or premature stop codons, and are therefore incapable of producing replication-competent virus [9]. It is the small fraction of genetically intact, latent proviruses that constitutes the true therapeutic target. This distinction has profound implications for interpreting HDACis clinical trial results: increases in cell-associated HIV RNA following HDACis treatment may disproportionately reflect transcription from defective proviruses, not the intact reservoir [18].

Moreover, the reservoir is not static. Clonal expansion of HIV-infected CD4+ T cells, driven by homeostatic proliferation and antigen-driven stimulation, means that eliminating individual infected cells may be counterbalanced by proliferation of clonal descendants [10]. This mechanism implies that even an effective LRA-mediated ‘shock’ could fail to reduce overall reservoir size if clonal expansion is ongoing.

These insights necessitate a reassessment of the ‘shock and kill’ strategy and highlight the need for LRA designs that achieve selective, sustained activation of intact proviruses, paired with robust immune or pharmacological ‘kill’ mechanisms.

### Tissue Reservoirs and Myeloid Cell Reservoirs

The HIV latent reservoir is not confined to resting CD4+ T cells in peripheral blood. Mounting evidence demonstrates that HIV establishes anatomically compartmentalized latent reservoirs in a diverse array of tissues and cell types, each presenting distinct barriers to eradication that extend well beyond those encountered in circulating lymphocytes. As Denton et al. have highlighted, peripheral blood reservoir measurements may not accurately reflect the size or composition of reservoirs in tissue compartments, underscoring the need for cure strategies that address the full anatomical scope of HIV persistence [19].

The principal tissue reservoirs include the gut-associated lymphoid tissue (GALT), lymph nodes, the Central Nervous System (CNS), and the reproductive tract. HIV DNA levels in the gut are consistently higher than those in peripheral blood, underscoring the importance of the gastrointestinal tract as a major viral sanctuary site [20]. Within lymph nodes, follicular helper T cells residing in germinal centers constitute a pharmacologically privileged reservoir, as the architecture of germinal centers limits both drug penetration and immune effector access [19]. CD4+ T cells within GALT also differ substantially in their phenotypic and differentiation characteristics from their circulating counterparts, and the mechanisms sustaining HIV persistence in this compartment remain incompletely understood [20].

The CNS represents perhaps the most clinically consequential tissue reservoir. Microglial cells, the brain-resident macrophages, are the principal HIV-infected cell type in the CNS, harboring integrated HIV DNA in long-lived cells through mechanisms that allow viral persistence for extended periods. Astrocytes also contribute to CNS viral persistence, with up to 10 to 20% carrying HIV DNA in the cell genome [13]. The CNS reservoir is particularly resistant to LRA-based strategies for two reasons. First, the blood–brain barrier restricts the penetration of most pharmacological agents into the CNS. Second, in a clinical substudy of the CLEAR panobinostat trial [21], neither panobinostat nor HIV RNA was detected in the cerebrospinal fluid of any of the 11 participants assessed, indicating that panobinostat either does not penetrate the CNS or reaches concentrations below the limit of detection at standard clinical dosing. Vorinostat and romidepsin have been shown in preclinical studies to cross the blood–brain barrier to a limited degree, though whether the CNS concentrations achieved are sufficient to reactivate microglial or astrocytic reservoirs remains uncertain [22]. This means that the CNS reservoir is likely to remain largely untouched by the HDACis currently evaluated in clinical trials, even when robust latency reversal is achieved in peripheral blood CD4+ T cells.

Beyond the CNS, myeloid cells, including monocytes and tissue macrophages, constitute a distinct and increasingly recognized reservoir compartment. Using a monocyte-derived macrophage quantitative viral outgrowth assay (MDM-QVOA) and a myeloid-adapted intact provirus DNA assay, Veenhuis et al. (2023) demonstrated that monocyte-derived macrophages harbor replication-competent latent HIV in virologically suppressed patients, with intact proviral genomes detectable in 40% of participants, and that virus produced from these cells was capable of infecting bystander cells [12]. The contribution of myeloid reservoirs to viral rebound after treatment interruption thus warrants serious consideration in cure strategies. Importantly, myeloid cells differ mechanistically from CD4+ T cells in their latency biology: they are non-dividing, terminally differentiated cells with distinct chromatin accessibility profiles and are not susceptible to the TCR-mediated reactivation signals that can be exploited in T cell models [23].

The implications for HDACis-based LRA strategies are significant. In latently infected monocyte-derived macrophage models, vorinostat has been shown to reverse the latency restriction, whereas panobinostat notably failed to do so, suggesting that isoform selectivity and structural class matter considerably when targeting myeloid reservoirs [23]. Furthermore, HIV isolated from CNS compartments shows significantly reduced transcriptional responses to LRAs, including panobinostat and romidepsin, compared with lymphoid-derived virus isolated from the same patients, attributed to CNS-specific sequence variation in the LTR that alters transcription factor binding [24]. These findings collectively reinforce the argument that a one-size-fits-all LRA approach based on peripheral blood CD4+ T cell data is insufficient, and that anatomical compartment-specific pharmacokinetics and proviral biology must be factored into future HDACis design.

Taken together, these findings indicate that achieving HIV eradication will require LRA strategies capable of reaching and reactivating anatomically diverse reservoirs, including those in immune-privileged sites with limited drug penetration. Future design programs for HDACis should consider CNS penetrance as a key pharmacokinetic parameter alongside the established goals of class I isoform selectivity and metabolic stability. Combination strategies that pair systemically active HDACis with CNS-penetrant agents, or that employ targeted drug delivery approaches to bypass the blood–brain barrier, represent directions that merit further investigation.

## 4. The ‘Shock and Kill’ Strategy: Current State and Conceptual Limitations

‘Shock and kill’ posits that pharmacological reactivation of latent HIV, combined with cART to block new infections, exposes reactivated cells to immune-mediated killing by HIV-specific CD8+ cytotoxic T lymphocytes (CTLs) and natural killer (NK) cells [25]. The appeal of this approach is its mechanistic clarity; however, clinical trials have consistently failed to translate ex vivo latency reversal into measurable reservoir reduction [26,27].

Several mechanistic explanations have been advanced: First, HDACis, particularly pan-HDAC inhibitors, have immunomodulatory effects that may impair the very effector cells needed for the ‘kill’ phase. Vorinostat treatment has been shown to reduce cytokine production by HIV-specific T cells and suppress NK cell function [11]. Second, the pace of viral protein expression following LRA-mediated reactivation may be insufficient to trigger CTL recognition before the infected cell returns to latency. Third, and perhaps most fundamentally, the intact provirus burden is so small that detectable reservoir reduction may require years of continuous LRA exposure, which is a standard that current agents cannot meet, given the combination of insufficient potency, poor tolerability, and inability to sustain durable target engagement at clinically acceptable doses [28].

### Alternative Strategies: ‘Block and Lock’

In recognition of the limitations of shock and kill, the ‘block and lock’ strategy has emerged as a conceptual alternative. Rather than reactivating latent virus, block and lock aims to promote permanent, irreversible silencing of the integrated provirus, achieving a ‘functional cure’ in which the virus is permanently suppressed even in the absence of cART. Latency-promoting agents (LPAs) are the pharmacological tools of this strategy: they must induce epigenetic modifications at the HIV-1 LTR that are durable enough to resist cellular activation signals after compound withdrawal [29].

The most extensively studied LPA is didehydro-cortistatin A (dCA), a highly potent inhibitor of the HIV Tat protein. By blocking the Tat-TAR interaction, dCA disrupts the Tat-driven transcriptional amplification feedback loop, which over time promotes heterochromatin formation at the LTR through increased nucleosomal occupancy at Nucleosome-1 and reduced RNA polymerase II recruitment [30,31].

In the bone marrow-liver-thymus (BLT) humanized mouse model, dCA combined with cART suppressed HIV rebound for up to 19 days after treatment interruption, providing preclinical proof-of-concept for the block-and-lock approach [32]. However, as of 2024, no LPA including dCA has achieved permanent, irreversible proviral silencing in any model system, and none has entered clinical trials, underscoring the substantial translational gap that remains [29].

Epigenetic Drug Counterparts to HDACis in Block and Lock

While HDACis promote transcriptional activation by preventing histone deacetylation, the epigenetic counterparts relevant to block and lock are agents that enforce the repressive chromatin marks responsible for proviral silencing. Three principal classes of epigenetic targets have been identified at the HIV-1 LTR, each representing a distinct therapeutic opportunity.

The first and most studied target is the Polycomb Repressive Complex 2 (PRC2), which deposits the repressive histone mark H3K27me3 at the LTR through its catalytic subunit EZH2. H3K27me3 is one of the primary silencing marks on latent proviruses in primary CD4+ T cells, and its removal by the demethylase UTX/KDM6A is associated with proviral reactivation [33]. Conversely, pharmacological inhibition of UTX with the dual JMJD3/UTX inhibitor GSK-J4 enhanced H3K27me3 levels at the HIV LTR, suppressed reactivation of latent HIV in patient-derived cells, and increased LTR DNA methylation, suggesting a reinforcing relationship between histone and DNA methylation at the proviral promoter [33]. From a medicinal chemistry perspective, EZH2 activators or UTX inhibitors, rather than the EZH2 inhibitors used in oncology, represent a rational design direction for block-and-lock agents targeting this pathway. The SAR challenge is to identify molecules that selectively enhance H3K27me3 deposition at integrated retroviral sequences without broadly disrupting cellular Polycomb-regulated gene expression.

The second target is the H3K9 methyltransferase EHMT2 (also known as G9a), which deposits the repressive H3K9me2 mark at the LTR and cooperates with PRC2 to maintain proviral silencing. Chromatin immunoprecipitation studies have demonstrated that both EZH2 and EHMT2 are enriched at the 5′ LTR of latent proviruses and are rapidly displaced upon reactivation [34]. Importantly, CRISPR-mediated knockout of EZH2 depletes both H3K27me3 and H3K9me2, suggesting that the two repressive marks are mechanistically coupled at the proviral promoter. Pharmacological agents that activate or stabilize EHMT2 activity at the LTR, therefore, represent a complementary block-and-lock strategy to PRC2 targeting. Like EZH2 modulators, the selectivity challenge here is formidable: EHMT2 regulates a broad transcriptional programme in resting T cells, and systemic EHMT2 activation could have pleiotropic consequences. Integration site-aware or nanobody-based targeting approaches may ultimately be required to achieve provirus-selective silencing via this pathway.

The third relevant target is DNA methylation. CpG islands flanking the HIV-1 LTR are subject to methylation by DNA methyltransferases (DNMTs), and hypermethylation of these sites is associated with deep, stable latency that is resistant to conventional LRA-mediated reactivation [33]. While DNMT inhibitors such as 5-azacytidine act as LRAs in the shock-and-kill context by demethylating the LTR, the inverse approach, using DNMT-recruiting or DNMT-activating agents to enforce LTR hypermethylation, is an underexplored avenue for block and lock. The interdependence of H3K27me3 and DNA methylation observed by Nguyen et al. [33] suggests that agents targeting either mark may synergistically reinforce the other, opening the possibility of combination epigenetic block-and-lock regimens.

HDACis, Chromatin State, and Rational Combination Design

**Figure 1:**
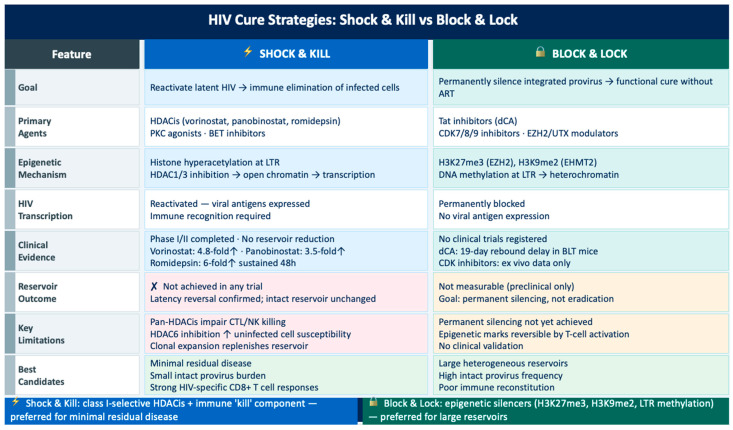
Visual comparison of the ‘shock and kill’ and ‘block and lock’ HIV cure strategies across eight features: goal, primary agents, epigenetic mechanism, HIV transcription, clinical evidence, reservoir outcome, key limitations, and best candidate patients. LRA, latency-reversing agent; LPA, latency-promoting agent; HDACis, histone deacetylase inhibitors; CTL, cytotoxic T lymphocyte; NK, natural killer cell; bNAb, broadly neutralizing antibody; dCA, didehydro-cortistatin A; LTR, long terminal repeat. Symbols: →: leads to/sequential process; ↑: increase; ✕: outcome not achieved in any trial.

Although HDACis do not directly contribute to block and lock, the chromatin states they characterize are directly informative for the design of LPAs. The repressive chromatin architecture at the HIV-1 LTR, maintained by HDAC1/3 recruitment and the resulting histone hypoacetylation, is the starting state that block-and-lock agents must preserve and reinforce. A rational combination design approach would pair a class I-selective member of the HDACis at a sub-reactivating dose, sufficient to define the baseline chromatin landscape without triggering LTR transcription, with an EZH2-stabilizing or EHMT2-activating agent and a DNMT-recruiting molecule, to cooperatively lock all three repressive marks simultaneously. Recent work identifying CDK8/19 inhibitors (Senexin A) and CDK7 inhibitors (YKL-5-124) as potent LPAs that block P-TEFb-dependent HIV transcriptional elongation without activating the LTR [35] adds a fourth mechanistically orthogonal component to such a combination. Block and lock may therefore ultimately require a multi-agent epigenetic cocktail analogous in principle to the cART regimens that transformed HIV treatment, with each agent targeting a distinct node of the proviral silencing network. The two strategies, shock and kill and block and lock, may ultimately be complementary: block and lock applied to patients with large, heterogeneous reservoirs to prevent rebound, and shock and kill reserved for patients with minimal residual disease where the intact provirus burden is sufficiently small to be targeted effectively, Figure 1 [36,37].

## 5. HDAC Biology and the Pharmacophore Framework

Human HDACs comprise 18 isoforms grouped into four classes based on phylogenetic similarity and co-factor dependence. Class I (HDAC1, 2, 3, 8), class II (HDAC4–7, 9, 10), and class IV (HDAC11) are zinc-dependent metalloenzymes and are the targets of all clinically relevant HDACis discussed herein. Class III enzymes (sirtuins) are NAD+-dependent and are structurally and pharmacologically distinct [38].

Zinc-dependent HDACs catalyze the removal of acetyl groups from ε-N-acetyl lysine residues on histone tails, promoting chromatin condensation and transcriptional repression. At the HIV-1 LTR, HDAC1 and HDAC3 are directly recruited by the NF-κB repressor complex and by the Tat co-repressor CTIP2, maintaining proviral silencing [14,39]. HDACis disrupt this process by chelating the catalytic zinc ion, preventing deacetylation, and driving histone hyperacetylation, favoring an open chromatin state that permits transcription factor access to the LTR [15].

### The Three-Component Pharmacophore

**Figure 2:**
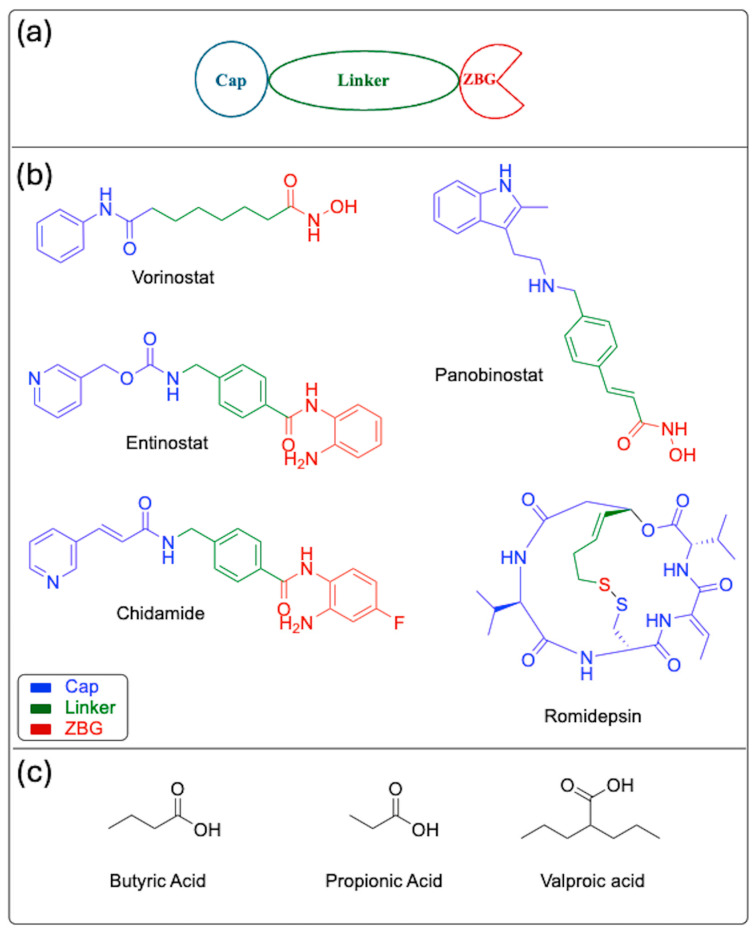
(**a**) Pharmacophore of HDACis. (**b**) Structures of HDACis of major classes (Hydroxamic acid, Benzamides, and Cyclic peptides). (**c**) Structures of HDACis of Short-Chain Fatty Acids class.

Virtually all zinc-dependent HDACis share a conserved three-component pharmacophore [40], Figure 2a:

- Zinc-binding group (ZBG): Chelates the catalytic Zn^2+^ ion in the active site. Determines potency and influences isoform selectivity.
- Linker region: Mimics the lysine side chain extending from the ZBG through the tubular hydrophobic channel of the active site. Length, rigidity, and geometry critically influence isoform engagement.
- Capping group (surface recognition domain): Interacts with residues at the rim of the HDAC active site. Capping group is the primary determinant of isoform selectivity, as rim residues vary substantially across isoforms.

Understanding this pharmacophore is central to rational HDACis design and to interpreting the differential isoform selectivity profiles that underlie the distinct clinical phenotypes of each drug class.

## 6. Structural Classes of HDAC Inhibitors: Chemistry, SAR, and HIV Latency Reversal

Table 2 provides a comparative SAR summary of representative HDACis across all four structural classes. EC50 values should be interpreted with caution: they vary substantially depending on the assay system (biochemical enzyme assay vs. cell-based reporter vs. primary cell HIV RNA induction), the cell model used, and the endpoint measured. Where possible, the assay type, cell model, and endpoint are specified in the table. Readers are encouraged to consult the primary sources cited for full experimental details.

### 6.1. Hydroxamic Acids

The hydroxamic acid (–C(=O)NHOH) moiety is the most widely exploited ZBG in HDACis development owing to its bidentate coordination of the active-site zinc ion via both the carbonyl oxygen and the hydroxyl nitrogen. This strong metal affinity confers sub-micromolar to nanomolar potency across class I and II HDACs, but at the cost of broad-spectrum activity and the potential for off-target inhibition of other zinc-dependent metalloenzymes, including matrix metalloproteinases (MMPs) and carbonic anhydrase [53].

Vorinostat (SAHA)

Vorinostat exemplifies the minimal pharmacophore of a hydroxamic acid HDACis: a flexible aliphatic six-carbon linker connecting the hydroxamic acid ZBG to a phenyl cap with an amide anchor, Figure 2b. This simple architecture provides excellent aqueous solubility and broad HDAC inhibition (IC50 10–20 nM for HDAC1/2/6) but limited isoform discrimination [41].

In the landmark Archin et al. (2012) [27] clinical trial, a single oral dose of vorinostat (400 mg) produced a significant, though transient, increase in HIV RNA expression in resting CD4+ T cells in patients on suppressive cART. Subsequent interval-dosing trials confirmed latency reversal but demonstrated no statistically significant reduction in the reservoir [27,43]. From an SAR perspective, the principal limitations of vorinostat as an LRA are its short plasma half-life (approximately 2 h) and rapid glucuronidation, which limit sustained HDAC inhibition at the LTR. Structural modifications to the linker, introduction of rigidity via trans-double bonds or aromatic rings, and to the cap group (e.g., replacement of the phenyl ring with pyridyl or indolyl systems) have been explored to improve pharmacokinetics, though few analogues have entered clinical evaluation for HIV [54].

A further and clinically significant off-target liability of vorinostat was identified by Lucera et al. (2014) [55]. In addition to reactivating latent HIV, vorinostat significantly increases the susceptibility of uninfected CD4^+^ T cells to HIV infection in a dose- and time-dependent manner [55]. This effect operates not through enhanced viral fusion or receptor upregulation, but through acceleration of post-entry events, specifically reverse transcription, nuclear import, and proviral integration, within previously uninfected bystander cells, raising the concern that vorinostat may reseed the reservoir it is intended to purge, particularly under conditions of suboptimal cART exposure. Crucially, selective inhibition of the cytoplasmic class IIb enzyme HDAC6 with the isoform-selective probe tubacin recapitulated this pro-infectious phenotype, directly implicating HDAC6, rather than nuclear class I HDACs, as the driver of this adverse effect [55]. This finding carries important SAR implications for hydroxamic acid HDACis design. The pan-HDAC inhibitory profile of vorinostat, which encompasses cytoplasmic HDAC6 alongside nuclear HDAC1/2/3, appears to be the source of its pro-infectious liability. HDACis engineered to selectively inhibit nuclear class I HDACs while sparing HDAC6 would be predicted to retain LTR-reactivating potency without augmenting bystander cell susceptibility to HIV acquisition. From a medicinal chemistry standpoint, this selectivity goal is addressable through several structural strategies: (i) adoption of the 2-aminobenzamide ZBG in place of the hydroxamic acid moiety, since benzamides are inherently disfavored by HDAC6 owing to steric incompatibility with its wider active-site channel; (ii) shortening of the linker relative to the six-carbon chain of SAHA to reduce engagement of the deeper HDAC6 catalytic pocket; and (iii) introduction of sterically demanding cap groups that interact with class I-specific rim residues while creating steric clashes with HDAC6 surface topology [56]. These considerations substantially reinforce the rationale, developed further in Section 6.2, for prioritizing class I-selective benzamide analogues over pan-hydroxamic acid inhibitors in future HIV LRA programs.

Panobinostat

Panobinostat introduces a longer, more conformationally flexible linker incorporating an indolyl-acryloyl unit that extends the reach of the ZBG deeper into the HDAC active site, Figure 2b. This structural feature correlates with significantly enhanced potency (EC50 ~2–5 nM ex vivo) but also increased cytotoxicity compared to vorinostat [21]. Phase I/II clinical trials demonstrated that panobinostat produced a mean 3.5-fold increase in plasma HIV-1 RNA in patients on suppressive cART, a greater response than vorinostat, but again without reservoir reduction.

A critical and mechanistically important finding from a 2024 study by Armani-Tourret et al. is that panobinostat treatment selects for proviruses with specific epigenetic features that render them resistant to reactivation—so-called ‘epigenetically privileged’ proviruses that were enriched in the reservoir following treatment [44]. This represents an under-appreciated risk of pan-HDAC inhibition; therapeutic pressure may not merely fail to reduce the reservoir but could actively reshape it towards a more refractory composition. The voluntary withdrawal of Farydak from the US market by Novartis in 2022, which was a commercial decision rather than a new safety signal, has further limited its development for HIV indications.

From an SAR standpoint, the selectivity problem of pan-hydroxamic acid inhibitors has driven interest in structural modifications to the linker and cap group. Introduction of bulkier, more sterically demanding cap groups can introduce isoform selectivity, though the strong zinc-chelating potency of the hydroxamic acid ZBG tends to override cap-group contributions to selectivity at high concentrations [53,54].

### 6.2. Benzamides

Benzamide HDACis are distinguished by a 2-aminobenzamide ZBG that chelates the active-site zinc in a bidentate fashion through the amide carbonyl and the ortho-amino group. This binding mode is structurally incompatible with the active sites of class IIa HDACs (due to differences in a key ‘gatekeeper’ tyrosine/histidine residue) and consequently confers intrinsic Class I (HDAC1/2/3) selectivity, which presents a substantial advantage over hydroxamic acids for applications where selective inhibition of the nuclear, transcription-regulatory HDACs is desired [57].

Mechanistically, benzamides exhibit slower ‘slow-binding’ kinetics characterized by rapid initial association followed by a conformational change in the HDAC active site that produces a tight, slowly reversible complex. This kinetic profile results in prolonged target engagement even at lower plasma concentrations, which may be favorable for sustained HIV-LTR reactivation [58].

Entinostat and Chidamide

Entinostat employs a pyridylmethyl cap group connected via a phenyl carbamate linker to the 2-aminobenzamide ZBG, Figure 2b. The pyridine nitrogen participates in a hydrogen bond with a rim residue of HDAC1 and HDAC3, contributing to isoform selectivity. Chidamide (tucidinostat), approved in China and several other countries for peripheral T-cell lymphoma, shares the 2-aminobenzamide pharmacophore with a benzamide cap modified to incorporate a fluorine substituent and an acrylamide linker, further favoring HDAC1/2/3 selectivity with reduced activity against HDAC6, Figure 2b, ref. [48].

Kobayashi et al. (2017) conducted a systematic comparison of HDACis in primary resting CD4+ T cells from HIV-infected individuals and identified benzamide-containing HDACis, particularly those with a pyridyl cap group, structurally similar to chidamide, as among the most potent and least toxic LRAs in this primary cell model [47]. This finding provides a strong SAR-based rationale for prioritizing class I-selective benzamides in future HIV LRA development. Critically, the selectivity for HDAC1/3 may also reduce immunosuppressive off-target effects relevant to NK and CTL function, though direct evidence for this in HIV models is limited and represents an important research gap.

### 6.3. Cyclic Depsipeptides

Romidepsin (FK228) is a naturally derived bicyclic depsipeptide produced by *Chromobacterium violaceum*. It is administered as a prodrug: the two disulfide bonds in the macrocyclic ring are reduced intracellularly to reveal two free thiol groups that serve as the ZBG, chelating the active-site zinc of class I HDACs with low-nanomolar affinity, Figure 2b [59].

The rigid bicyclic macrocyclic scaffold of romidepsin provides unique binding conformations that are structurally inaccessible to linear HDACis, resulting in a binding mode with extraordinary potency (IC50 < 1 nM for HDAC1/2) and an unusually sustained duration of action. In primary CD4+ T cells from HIV-infected donors on cART, romidepsin elicited a 6-fold increase in intracellular HIV RNA within four hours, and the activating effect was maintained for up to 48 h, which is substantially longer than the transient responses observed with vorinostat and panobinostat [45].

Romidepsin also demonstrated dose-dependent inhibition of de novo HIV infection in vitro [45], suggesting an additional virological benefit during latency reversal trials. However, Phase I/II clinical trials confirmed the now-familiar pattern: robust latency reversal without reservoir reduction [46]. Notably, romidepsin’s potent immunomodulatory effects, including significant immunosuppression at the doses required for HIV latency reversal, remain a key clinical challenge. Phase II dose-optimization and combination approaches (e.g., romidepsin + anti-HIV broadly neutralizing antibodies) are underway.

From an SAR perspective, the synthetic inaccessibility of the macrocyclic scaffold limits analogue exploration; however, synthetic cyclic tetrapeptide scaffolds (e.g., trapoxin derivatives) offer a tractable platform for building macrocyclic diversity with analogous binding modes and are an underexplored avenue for HIV LRA development [60].

### 6.4. Short-Chain Fatty Acids

Short-chain fatty acids (SCFAs), including butyrate, propionate, and valproic acid, shown in Figure 2c, inhibit class I and II HDACs through a non-chelating mechanism involving insertion of the carboxylate into the active-site channel and disruption of hydrophobic contacts. This binding mode is inherently weaker than hydroxamic acid or benzamide coordination of zinc, resulting in millimolar EC50 values for HIV latency reversal in primary cell models [50,61].

Butyrate, produced by microbial fermentation of dietary fiber, is of particular physiological interest. Gut-associated butyrate concentrations can reach levels sufficient for HDAC inhibition locally, and oral pathogen-derived butyrate in the oral cavity may similarly influence HIV reservoirs in mucosal tissues. Das et al. [50] demonstrated that butyrate activates P-TEFb and induces multiple histone modifications at the HIV-1 LTR, producing robust latency reversal in Jurkat and primary T-cell models [50,51].

Valproic acid, the first HDAC inhibitor to enter clinical trials for HIV eradication, served as an important proof-of-concept for pharmacological epigenome manipulation but failed to reduce the latent reservoir in large-scale trials [52]. Its poor pharmacokinetics as an HDAC inhibitor, requiring plasma concentrations in the epilepsy treatment range (50–100 µg/mL) to achieve partial HDAC inhibition, combined with hepatotoxicity concerns, preclude its clinical development as an LRA.

The principal value of SCFAs in the current landscape is as mechanistic probes and as components of combination LRA strategies targeting multiple latency-maintaining pathways simultaneously, rather than as standalone therapeutic agents.

## 7. Preclinical and Clinical Evidence

Table 3 summarizes the clinical performance of the major HDACis evaluated as HIV LRAs, integrating trial design, key outcomes, and observed adverse effects.

### 7.1. The Central Paradox: Latency Reversal Without Reservoir Reduction

Across all clinical trials of HDACis as LRAs, the same pattern has emerged: reproducible, statistically significant increases in cell-associated or plasma HIV RNA, confirming pharmacological latency reversal but no measurable reduction in the frequency of latently infected cells or resting cell-associated HIV DNA [26,27,46,62]. This paradox has several non-mutually exclusive explanations:

- Defective provirus activation: HDACis reactivate both intact and defective proviruses. Since >90% of proviruses are defective, the bulk of the HIV RNA signal detected clinically may originate from defective genomes, obscuring the true impact on the intact reservoir [9].
- Incomplete kill: Reactivated cells may not be recognized or eliminated rapidly enough by immune effectors before the LRA is cleared and the cells return to latency [28].
- Immunosuppression by LRAs: Pan-HDAC inhibitors may suppress CTL and NK cell function precisely when immune killing is required [11].
- Clonal replacement: Homeostatic proliferation replenishes reservoir size even when individual infected cells are cleared [10].
- Selection pressure: Panobinostat treatment has been shown to enrich for epigenetically refractory proviruses [44].

These data collectively argue that LRA monotherapy is unlikely to achieve reservoir reduction, and that future trial designs must incorporate a potent, simultaneous ‘kill’ mechanism, such as anti-HIV broadly neutralizing antibodies (bNAbs), therapeutic vaccines, or NK cell-enhancing agents, alongside LRA administration.

### 7.2. The Immunosuppression Paradox

A critical concern specific to pan-HDAC inhibitors is their pleiotropic immunosuppressive activity. HDAC inhibition affects gene expression broadly, and clinical HDACis at doses required for HIV latency reversal have been shown to reduce IFN-γ production by HIV-specific T cells, suppress NK cell degranulation, and alter DC maturation [11]. Vorinostat, when administered to HIV-positive individuals on cART, produced measurable reductions in NK cell cytotoxicity. This property directly undermines the ‘kill’ component of shock and kill. Critically, evidence from longitudinal studies confirms that HIV-specific T cells do remain capable of recognizing infected cells during suppressive cART [63], making their preservation during LRA treatment essential and HDACis-mediated suppression of this function a meaningful clinical liability.

Isoform-selective agents, particularly class I-selective benzamides, may circumvent this problem. Walker-Sperling et al. (2016) demonstrated that different LRAs have divergent effects on primary CD8+ T cell function, with some LRAs preserving cytolytic capacity while others significantly impaired it [64]. Clutton and Jones (2018) further showed that these immune effects are highly LRA-specific and cannot be extrapolated across drug classes [65]. HDAC6-selective inhibitors have been shown to enhance rather than suppress immune cell function, suggesting that isoform selectivity profiling against HDAC6 is an important consideration [66]. The identification of HDAC isoforms that specifically regulate HIV-LTR chromatin versus those that regulate immune effector gene expression is therefore a critical research priority, and the answer will directly inform which selectivity profiles are most desirable for future HDACis-based LRAs [47,48].

## 8. Challenges and Opportunities

### 8.1. Isoform Selectivity as a Guiding Design Principle

The field has increasingly moved towards recognizing that pan-HDAC inhibition, while experimentally convenient, may be clinically suboptimal for HIV LRA applications. A desirable selectivity profile for a next-generation HDACis LRA would favor inhibition of isoforms responsible for HIV-LTR silencing (principally HDAC1 and HDAC3, which are recruited by NF-κB repressor complexes and CTIP2) while sparing isoforms required for immune effector function. Class I-selective benzamides, and next-generation isoform-selective molecules identified through structure-based drug design, represent among the more promising avenues towards this goal, though robust clinical validation is still lacking [47,57].

### 8.2. Pharmacokinetic and ADMET Limitations

All four structural classes of HDACis face distinct pharmacokinetic challenges as LRAs. Hydroxamic acids undergo rapid glucuronidation and hydrolysis, limiting plasma half-life. Benzamides display slow-onset kinetics that may require extended dosing intervals. Cyclic depsipeptides like romidepsin are expensive to manufacture and require intravenous administration. SCFAs are rapidly metabolized and require millimolar concentrations for HDAC inhibition. Computational ADMET prediction, integrating molecular docking, molecular dynamics, and quantitative SAR (QSAR) models, is an increasingly important tool for identifying analogues with improved pharmacokinetic profiles before synthesis [67].

### 8.3. Combination LRA Strategies

Combination LRA strategies targeting multiple latency-maintaining pathways simultaneously have demonstrated synergistic latency reversal in ex vivo models. Particularly promising combinations include HDACis + PKC agonist (e.g., bryostatin-1) and HDACis + BET bromodomain inhibitor (e.g., JQ1), which engage complementary transcriptional regulatory nodes [68,69]. Critically, combinations that pair LRAs with immune enhancers have begun to show preclinical proof-of-concept: Lu et al. (2016) demonstrated enhanced clearance of HIV-infected cells with romidepsin + broadly neutralizing antibodies (bNAbs) in vivo [70], and IL-15 superagonists that boost CTL responses represent another promising ‘kill’ component [71]. Chidamide has recently demonstrated direct anti-HIV activity in primary CD4+ T cells by upregulating p21 expression [49], further motivating its development as a benzamide LRA candidate in combination regimens.

### 8.4. Reservoir Heterogeneity and Intact Provirus Quantification

The near-exclusive focus of earlier clinical trials on bulk HIV DNA or total HIV RNA as endpoints has been shown to be misleading. Clonally expanded CD4+ T cells can produce replication-competent HIV-1 in vivo [72], meaning that bulk DNA measurements fail to distinguish persistent clonal proviruses from genuinely silenced reservoir cells. Quantitative viral outgrowth assays (QVOAs) and, more recently, the intact provirus DNA assay (IPDA) enable direct quantification of the intact, replication-competent reservoir [9]. Rasmussen and Lewin (2016) have argued that the field will not be able to accurately judge LRA efficacy without widespread adoption of these more sensitive and specific reservoir measurements [73]. Future clinical trials of HDACis must therefore mandate IPDA or QVOA endpoints to determine whether any impact on the true therapeutic target, the intact reservoir, is achieved.

## 9. Future Directions

### 9.1. Computational Drug Design and Next-Generation HDACis

The high-resolution crystal structures of HDAC1, HDAC2, and HDAC3 in complex with class I-selective inhibitors provide an excellent structural basis for structure-based drug design [39]. Molecular docking campaigns against these templates, combined with pharmacophore-based virtual screening of commercial and in-house compound libraries, represent an efficient strategy for identifying novel ZBG and cap group combinations with improved selectivity. Molecular dynamics (MD) simulations are particularly valuable for assessing the stability of the slowly reversible benzamide binding mode and for identifying cryptic allosteric binding sites that may be exploited for isoform selectivity.

Future medicinal chemistry efforts could explore: (i) synthesis of a series of 2-aminobenzamide analogues with systematically varied linker geometry (saturated, unsaturated, aromatic, and heterocyclic) to map the structural determinants of HDAC1 vs. HDAC3 selectivity; (ii) introduction of metabolically stabilizing substituents (e.g., deuterium, fluorine, methyl groups) to extend plasma half-life; and (iii) evaluation of selected compounds in primary CD4+ T cell latency models and ex vivo assays using cells from HIV-positive donors on suppressive cART, prioritizing IPDA-based endpoint assessment.

### 9.2. Novel Zinc-Binding Groups

The hydroxamic acid ZBG, despite its limitations, remains the benchmark for HDAC-binding potency. Non-hydroxamic ZBGs, including trifluoromethyl ketones, α-ketoamides, N-formyl hydroxylamines, and recently described triazole-based groups, have been explored as alternatives with improved zinc-binding selectivity profiles and reduced susceptibility to glucuronidation [54]. Of particular interest are nitrogen-containing heterocyclic ZBGs that adopt unique binding geometries in the HDAC active site, potentially enabling isoform selectivity not achievable with hydroxamic acids.

### 9.3. Combination Strategies and Clinical Trial Design

An emerging view in the field, formalized in the IAS Global Scientific Strategy for HIV Cure [74], holds that HDACis monotherapy is unlikely to reduce the HIV reservoir on its own. Future clinical trial designs should consider: (i) class I-selective HDACis as the LRA component; (ii) a co-administered immune activator or bNAb as the ‘kill’ component; (iii) endpoint assessment using IPDA or QVOA rather than total HIV DNA; and (iv) patient stratification by reservoir size and intact provirus frequency at baseline to identify the population most likely to benefit. Early phase combination trials have already evaluated romidepsin + 3BNC117 (ROADMAP, NCT02850016) and vorinostat + VRC07-523LS (VOR-07, NCT03803605), and both have been completed; neither demonstrated measurable reservoir reduction, though they established important safety and feasibility data [70,71]. Preclinical data support the rationale for this approach. Borducchi et al. demonstrated in SIV-infected rhesus macaques that Ad26/MVA therapeutic vaccination combined with TLR7 stimulation significantly delayed viral rebound after cART interruption, providing a proof-of-concept framework for pairing LRAs with immune activators [75]. The systematic review by Passaes and Sáez-Cirión underscores the importance of combining immunological and virological readouts in future cure-focused trial designs [76].

### 9.4. Pharmacovigilance and Long-Term Safety

The chronic nature of HIV infection and the potential for repeated LRA administration cycles mean that long-term safety monitoring is essential. Of particular concern are cardiovascular effects (QTc prolongation with romidepsin and panobinostat) and hematological toxicity (thrombocytopenia with vorinostat) [21,43,45,46]. A further concern is the selective enrichment of epigenetically privileged, treatment-resistant proviruses following repeated HDACis exposure. As observed with panobinostat, treatment preferentially reactivated proviruses integrated in chromatin regions with high H3K27ac marks, which are the accessible targets of HDACis activity. This reactivation-and-elimination selectively depleted the more accessible proviral population, leaving behind a residual reservoir disproportionately enriched for integration sites in heterochromatin-associated ZNF gene regions with reduced H3K27ac marks. These surviving proviruses are intrinsically less susceptible to HDACis-mediated reactivation, meaning that repeated LRA exposure may inadvertently create a pharmacological selection pressure that makes the residual reservoir progressively harder to target with subsequent treatment [44]. It is important to note that this concern is distinct from the intended on-target mechanism of HDACis, which is LTR histone hyperacetylation driving viral gene expression, the therapeutic goal of the shock-and-kill strategy. Systematic pharmacovigilance programs, integrated into future clinical trials from the outset, are necessary to characterize these risks.

### 9.5. Key Research Priorities and Open Questions

- Can benzamide-class HDACis be structurally optimized to achieve sustained plasma half-life and durable LTR engagement without the immunosuppressive off-target effects associated with pan-HDAC inhibition?
- How can the pro-infectious off-target effect of pan-HDAC inhibitors on uninfected CD4+ T cells, mediated through HDAC6 inhibition, be eliminated through structural modification while preserving LTR-reactivating potency?
- Do HDACis selectively reactivate intact, replication-competent proviruses, or do they predominantly activate defective proviruses, and how can future trial designs use the intact provirus DNA assay (IPDA) to answer this question definitively?
- What combination of LRA and immune-activating or killing agents is required to achieve measurable reduction of the intact HIV reservoir, and which patient populations—stratified by reservoir size, intact provirus frequency, and CD8+ T cell function—are most likely to benefit?
- Can rationally combined epigenetic regimens that pair class I-selective HDACis with histone methyltransferase stabilizers and DNA methylation-enforcing agents achieve durable, irreversible proviral silencing consistent with a block-and-lock functional cure?

## 10. Conclusions

This narrative, structured review has examined HDAC inhibitors as HIV LRAs through a medicinal chemistry lens, synthesizing SAR, isoform selectivity, and clinical evidence across four structural classes. The consistent failure of clinical trials to translate pharmacological latency reversal into reservoir reduction demands a fundamental reassessment of both the ‘shock and kill’ strategy and the properties required of a well-designed HDACis LRA.

This analysis suggests three design priorities for next-generation HDACis LRAs: (i) a selectivity profile favoring class I isoforms (principally HDAC1/3) to maximize HIV-LTR reactivation while reducing immune effector impairment; (ii) improved pharmacokinetics enabling sustained target engagement; and (iii) chemical tractability permitting iterative SAR optimization. The benzamide class, with its intrinsic class I selectivity and encouraging potency in primary HIV latency models, offers a potentially useful foundation for future medicinal chemistry efforts, though direct clinical validation in HIV is still absent for most benzamide candidates.

Achieving HIV eradication will likely require HDACis to be deployed not as monotherapy LRAs but as components of rationally designed combination regimens that pair selective latency reversal with robust immune or pharmacological elimination of reactivated cells. Whether this combination approach will prove sufficient to overcome intact provirus heterogeneity and clonal reservoir expansion remains an open and important question. Realizing this goal will require close integration of structural chemistry, molecular virology, and clinical immunology, which is a multidisciplinary challenge that the field is increasingly, though not yet fully, equipped to meet.

## Figures and Tables

**Table 1:** Literature Search Strategy and Results.

Databases	PubMed/MEDLINE, Scopus, Web of Science, ClinicalTrials.gov, Cochrane Library
Date range	January 2010–March 2026
Primary search terms	HIV latency, HDAC inhibitors, latency-reversing agents, shock and kill, epigenetic HIV cure, histone deacetylase
Secondary terms	vorinostat, panobinostat, romidepsin, entinostat, chidamide, valproic acid, butyrate, short-chain fatty acids, HIV reservoir, CD4+ T cells, SAR, isoform selectivity, tissue reservoirs, myeloid cells, CNS.
Additional filters	English language; human or primary cell studies for clinical sections; in vitro accepted for mechanistic sections; review articles used for background only
Total records retrieved	~1240 (PubMed 780, Scopus 310, Web of Science 150)
After deduplication	~920 unique records
Included after screening	76 primary research articles, 6 clinical trial reports, 34 review articles cited for contextualization, 1 guidelines/databases (WHO 2024 Global HIV Update)
Exclusion criteria	Duplicate reports, conference abstracts without full data, non-peer-reviewed sources, studies exclusively on non-HIV cancer HDACis with no HIV-related data, articles outside the 2010–2026 date range unless foundational.

**Table 2:** Comparative SAR Summary of Major HDAC Inhibitor Classes in HIV Latency Reversal.

Compound	Class	ZBG	Linker/Cap	HDAC Selectivity	HIV Latency Reversal EC50 (Assay/Cell Model/Endpoint)	Verified Clinical Status (as of March 2026)
Vorinostat (SAHA)	Hydroxamic acid	Hydroxamic acid	Flexible aliphatic/Anilinyl	Pan-HDAC (Class I & II); IC50 ~10–20 nM HDAC1/2/6 (biochemical)	500 nM (0.5 µM)/Primary resting CD4+ T cells from cART-suppressed donors/Cell-associated unspliced HIV RNA induction; note: ~1–2 µM reported in some cell-line models. [27,41,42]	Phase I/II completed (NCT01319383, NCT02475915); no reservoir reduction; combination trials completed (NCT03803605, NCT03212989) [27,43]
Panobinostat	Hydroxamic acid	Hydroxamic acid	Long, flexible/Indolyl-acryloyl	Pan-HDAC (Class I, II, IV); IC50 < 10 nM HDAC1/2 (biochemical)	~2–5 nM/Ex vivo primary CD4+ T cells/Cell-assoc. unspliced HIV RNA (3.5-fold increase) [21]; note: values assay-dependent	Phase 1/2 completed (NCT01680094, CLEAR study); no further HIV-specific trials registered as of March 2026 [21,44]
Romidepsin	Cyclic depsipeptide	Thiol (unmasked intracellularly by reduction)	Rigid bicyclic macrocycle; no cap group analogue	Class I selective (HDAC1/2); IC50 < 1 nM (biochemical); minimal Class II activity	~1–10 nM/Primary resting CD4+ T cells from cART-suppressed donors/Intracellular HIV RNA (6-fold at 4 h, sustained 48 h) [45]	Phase 1/2 completed (NCT01933594/ACTG 5315; NCT02092116/REDUC; NCT02850016/ROADMAP Phase 2a with 3BNC117); no reservoir reduction in any trial [45,46]
Entinostat	Benzamide	2-Aminobenzamide (ortho-amino)	Pyridylmethyl/Phenyl carbamate	Class I selective (HDAC1/3); IC50 ~0.5–2 µM (biochemical); inactive vs. Class IIa	~0.5 µM/Jurkat T-cell latency model (J-Lat)/GFP reporter reactivation [47]; primary CD4+ T cell data limited	No HIV-specific clinical trials registered; under preclinical investigation as LRA; approved for oncology indications in some jurisdictions [47,48]
Chidamide (Tucidinostat)	Benzamide	2-Aminobenzamide (ortho-amino)	Fluorinated benzamide cap/Acrylamide linker	Class I selective (HDAC1/2/3); HDAC6 sparing	~0.1–0.5 µM/Primary CD4+ T cells/p21 upregulation and HIV-1 inhibition assay [49]; HIV LRA EC50 not yet formally established in primary latency models	Approved in China, South Korea, and other markets for peripheral T-cell lymphoma; HIV use remains preclinical as of March 2026 [49]
Butyrate (Sodium butyrate)	Short-chain fatty acid	Carboxylate (non-chelating)	Short n-butyl chain	Class I & II (weak, non-selective); effective concentration ~mM range	~1–5 mM/Jurkat J-Lat and primary CD4+ T cells/P-TEFb activation and histone acetylation at LTR [50,51]; assay-dependent; not compared in primary reservoir models	Preclinical only; physiological concentrations relevant to gut-associated mucosal reservoir; not in clinical HIV trials [50,51]
Valproate (VPA)	Short-chain fatty acid	Carboxylate (non-chelating)	Branched 2-propyl pentanoate chain	Class I & II (weak); effective HDAC inhibition requires mM concentrations	~0.5–2 mM/Primary CD4+ T cells/HDAC inhibition and HIV RNA induction [52]; millimolar concentrations required, limiting therapeutic window	Phase I/II completed; failed to reduce reservoir in large-scale clinical trials; not being advanced further as HIV LRA [52]

**Table 3:** Clinical Trial Summary of HDAC Inhibitors as HIV LRAs (with Trial Identifiers, Participant Numbers, Dosing, Endpoints, and Citations; Status Verified March 2026).

Agent	Trial ID/Phase/N	Dosing Regimen	Primary Endpoint	Key Outcome	Reservoir Reduction	Notable Adverse Effects	Citation
Vorinostat (SAHA)	NCT01319383 (UNC); Phase I/II; *n* = 16 (single-dose arm); N = 20 (interval-dosing arm)	400 mg oral single dose; then 400 mg every 24 h × 3 days/week × several weeks	Change in cell-assoc. HIV RNA in resting CD4+ T cells (primary); plasma HIV RNA; reservoir size (QVOA)	Significant increase in cell-assoc. HIV RNA after single dose and interval dosing confirmed in resting CD4+ T cells; short-lived effect (~24 h per dose)	No statistically significant reduction in resting cell-associated HIV DNA or QVOA-measured reservoir	Fatigue, nausea, diarrhea, thrombocytopenia (grade 1–2); no dose-limiting toxicities	[27,43]
Panobinostat	NCT01680094 (CLEAR); Phase 1/2; *n* = 15	20 mg oral 3× per week every other week × 8 weeks, with continued cART	Change from baseline in cell-assoc. unspliced HIV RNA; secondary: plasma HIV RNA, total HIV DNA, IUPM	Median 3.5-fold increase (range 2.1–14.4) in cell-assoc. unspliced HIV RNA at all timepoints (*p* < 0.0001); transient plasma viraemia in all participants	No significant reduction in total HIV DNA or IUPM; Armani-Tourret et al. (2024) showed enrichment of epigenetically privileged proviruses post-treatment [44]	QTc prolongation, nausea, thrombocytopenia, fatigue; no active HIV-specific trials as of March 2026	[21,44]
Romidepsin	NCT01933594 (ACTG 5315); Phase 1/2; *n* = 20 | NCT02092116 (REDUC); Phase 1b/2a; *n* = 20 | NCT02850016 (ROADMAP); Phase 2a; *n* = 20 (randomized, with/without 3BNC117)	ACTG 5315: IV 5 mg/m^2^ day 1, 8, 15 of 28-day cycle; REDUC: IV 5 mg/m^2^ × 3 doses; ROADMAP: IV romidepsin + IV 3BNC117 (30 mg/kg) × 2 cycles	Safety and tolerability; activation of HIV-1 expression (cell-assoc. HIV RNA); reservoir size	6-fold increase in intracellular HIV RNA within 4 h; sustained effect to 48 h (ACTG 5315); ROADMAP: combination with 3BNC117 did not delay viral rebound during ATI	No significant reduction in HIV reservoir in any completed trial; ROADMAP specifically showed no IPDA or QVOA declines clearly exceeding assay variance	Nausea, fatigue, transient lymphopenia; QTc prolongation at higher doses; cardiac monitoring required; IV administration limits outpatient use	[45,46]
Valproic acid (VPA)	Phase I/II (multiple small trials); largest: *n* = 56	Oral VPA 250–750 mg twice daily (epilepsy-range dosing), with continued cART; duration 3–18 months across trials	Change in resting CD4+ T cell reservoir frequency (IUPM); proof-of-concept HDAC inhibition in vivo	Initial Lehrman et al. report showed ~75% decline in reservoir in 3/4 patients; not replicated in subsequent larger trials; Sagot-Lerolle et al. (*n* = 56) showed no significant effect	Failed to reduce reservoir in all adequately powered clinical trials; initial positive signal not reproduced	Hepatotoxicity (requires LFT monitoring), teratogenicity, GI intolerance, weight gain; not recommended for long-term HIV LRA use	[52]

## Data Availability

No new data were created or analyzed in this study.
